# Exercise-induced bronchoconstriction in university field hockey athletes: Prevalence, sex differences, and associations with dyspnea symptoms

**DOI:** 10.3389/falgy.2022.994947

**Published:** 2022-09-30

**Authors:** Robert S. Needham, Graham R. Sharpe, Neil C. Williams, Paul A. Lester, Michael A. Johnson

**Affiliations:** School of Science and Technology, Nottingham Trent University, Nottingham, United Kingdom

**Keywords:** exercise-induced bronchoconstriction, exercise-induced asthma, dyspnea, athletes, eucapnic voluntary hyperpnea, prevalence

## Abstract

**Introduction:**

Exercise-induced bronchoconstriction (EIB) is a prevalent condition in athletes. EIB screening studies identify many athletes with undiagnosed EIB. Moreover, there is a poor relationship between EIB and dyspnea symptoms recalled from memory.

**Purpose:**

This study investigated: (I) the prevalence of EIB in British university field hockey athletes; (II) the effect of sex and diagnostic criteria on EIB prevalence; and (III) the association between EIB and contemporaneous dyspnea symptoms.

**Methods:**

52 field hockey athletes (age: 20 ± 2 years; height: 173 ± 9 cm; body mass: 72 ± 10 kg; male = 31; female = 22) completed a eucapnic voluntary hyperpnea (EVH) test with multi-dimensional dyspnea scores measured 3–10 mins post-EVH. A test was deemed positive (EIB^+^) if a fall index (FI) ≥10% in FEV_1_ occurred at two consecutive time points post-test (FI_ATS_). Two further criteria were used to assess the effect of diagnostic criteria on prevalence: FI_≥10%_, determined by a pre-to-post-EVH fall in FEV_1_ of ≥10% at any single time-point; and FI_≥10%−NORM_ calculated as FI_≥10%_ but with the fall in FEV_1_ normalized to the mean ventilation achieved during EVH.

**Results:**

EIB prevalence was 19% and greater in males (30%) than females (5%). In EIB^+^ athletes, 66% did not have a previous diagnosis of EIB or asthma and were untreated. Prevalence was significantly influenced by diagnostic criteria (*P* = 0.002) ranging from 19% (FI_ATS_) to 38% (FI_≥10%−NORM_). Dyspnea symptoms were higher in EIB^+^ athletes (*P* ≤ 0.031), produced significant area under the curve for receive operator characteristics (AUC ≥ 0.778, *P* ≤ 0.011) and had high negative prediction values (≥96%).

**Conclusion:**

Overall, 19% of university field hockey athletes had EIB, and most were previously undiagnosed and untreated. EVH test diagnostic criteria significantly influences prevalence rates, thus future studies should adopt the ATS criteria (FI_ATS_). Contemporaneous dyspnea symptoms were associated with bronchoconstriction and had high negative prediction values. Therefore, contemporaneous dyspnea scores may provide a useful tool in excluding a diagnosis of EIB.

## Introduction

Exercise-induced bronchoconstriction (EIB) is characterized by a transient narrowing of the airways following exercise. In susceptible individuals EIB is triggered by increased airway surface osmolality and reduced airway temperature caused by exercise hyperpnea, and is characterized by airway smooth muscle contraction, inflammation, and hyperaemia (1). These processes reduce airway caliber and lung function which may limit athletic performance, particularly in endurance sports (2). Screening athletes for EIB is therefore important to understand the scale of the issue and ensure athletes who require treatment are identified.

The prevalence of EIB in athletes competing in individual (e.g. badminton, rowing, speed skating, athletics) and team sports (e.g. soccer, rugby, hockey, volleyball, lacrosse) is relatively high (18%–62%) (3–5), although it may vary depending on the type of sport and the associated ventilatory demand, and the environmental conditions in which the sport is performed. A lower prevalence of EIB (13%) has been reported in non-athletes, although this may be an underestimate because this cohort only included individuals without a prior diagnosis of asthma (6). Unfortunately, there is an increasing realization that self-reported respiratory symptoms yield a high number of both false-positive and false negative EIB diagnoses (3–5, 7). Indeed, previous reports on the poor diagnostic accuracy of respiratory symptoms may be undermined by diagnosis methodology. For instance, respiratory symptoms were assessed retrospectively through memory recall with participants at rest and asymptomatic (3–5), rather than shortly after a bronchial provocation test when EIB and symptoms are present. This is problematic because the reduction in forced expiratory volume in 1 s (FEV_1_), a hallmark characteristic of EIB, may relate poorly to respiratory symptoms that are assessed retrospectively and reliant on memory (8). Conversely, momentary conscious respiratory symptoms are more likely to relate to the severity and underpinning pathophysiology of EIB if assessed soon after a bronchial provocation test (8).

An EIB diagnosis is typically made by measuring changes in lung function shortly after a bronchoprovocation test such as exercise, EVH or inhaled mannitol. However, previous studies have used different diagnostic criteria which may affect EIB prevalence (3–6, 9). Furthermore, sex differences in the prevalence of EIB in athletes is rarely addressed. Given that EIB may compromise health and exercise performance it is important to establish the extent of the issue EIB presents in athletes and understand the influence of diagnostic criteria and sex on prevalence. Moreover, there is a need to understand the interplay between EIB and dyspnea symptoms that are assessed soon after a bronchoprovocation test.

Therefore, the aims of the present study were to investigate: (I) the prevalence of EIB in university field hockey athletes; (II) the effect of sex and diagnostic criteria on EIB prevalence; and (III) the relationship between EIB and contemporaneous dyspnea symptoms.

## Material and methods

### Participants

Fifty-three British university field hockey athletes (age: 20 ± 2 years; height: 173 ± 9 cm; body mass: 72 ± 10 kg; male = 31; female = 22), training and competing 8 ± 3 h per week and competing in British University Championships fixtures, provided written informed consent to participate in the study. The study was approved by the Nottingham Trent University Human Ethics Committee (approval number: 582) and all procedures conformed to the standard set by the Declaration of Helsinki.

### Experimental design

Testing took place across four consecutive months (November to February). Participants attended the laboratory on one occasion to perform an EVH test with spirometry measured before and after to evaluate the presence and severity of EIB. Ten minutes after the EVH test, participants completed a multidimensional dyspnea profile (MDP) (10). Participants refrained from exercise and caffeine ingestion on the day of testing. Participants with prescribed asthma medication (*n* = 4) were instructed to cease taking their medication prior to the EVH test (short-acting *β*_2_-agonist: ≥8 h; short-acting muscarinic antagonist: ≥4-h; long-acting bronchodilators (≥24-h; inhaled corticosteroids: ≥12-h; combined long-acting bronchodilators + inhaled corticosteroids: ≥24-h) (1, 11, 12).

#### Spirometry and EVH test

Baseline spirometry was assessed according to ATS/ERS guidelines (13) using a pneumotachograph (Pneumotach; Vitalograph, Buckingham, UK) calibrated with a 3-L syringe. EIB was assessed using an EVH test comprising 6-minutes of breathing at a target minute ventilation (${\dot{V}}_{E}$) of 85% of predicted maximal voluntary ventilation (MVV) (baseline FEV_1_ × 30) (12, 15). The EVH test was set up as previously described (12) and was considered valid if the mean ${\dot{V}}_{E}$ was ≥60% of predicted MVV (baseline FEV_1_ × 21), or a participant averaged <60% of predicted MVV whilst having a positive EVH test response. If a participant did not meet the criteria for a valid EVH test they were invited back for a second attempt. ${\dot{V}}_{E}$ was recorded from the volume of air passed through a dry gas meter every minute. Spirometry was assessed in duplicate 3, 6, 10, 15, and 20-min after the EVH test, with the highest values used for subsequent analysis. The difference between pre and post EVH test spirometry was termed the fall index (FI).

#### EIB diagnostic criteria

EIB was diagnosed when FEV_1_ fell, relative to baseline, by ≥10% at two consecutive time points after EVH, which conforms to ATS guidelines (hereafter termed FI_ATS_) (1, 14). Participants with a positive or negative FI_ATS_ EVH test response were classified as either EIB positive (EIB^+^) or EIB negative (EIB^−^). To evaluate the effect of diagnostic criteria on the prevalence of EIB, two additional criteria were used: (1) a pre-to-post-EVH fall in FEV_1_ of ≥10% at any single time-point (hereafter termed FI_≥10%_) (15); and (2) calculated the same as FI_≥10%_ but with the FI normalized to the mean ${\dot{V}}_{E}$ achieved during EVH (hereafter termed FI_≥10%−NORM_) (16) using the following calculation:

$$\left(\frac{\mathrm{Baseline}\;\mathrm{FE}V_{1}-\mathrm{Post}\;\mathrm{FE}V_{1}}{\mathrm{Baseline}\;\mathrm{FE}V_{1}})\times (\frac{30\times \mathrm{FE}V_{1}}{\mathrm{Acheived}\;{\dot{V}}_{E}}\right)$$

#### Multi-dyspnea profile

Between 3 and 10 mins after the EVH test [which typically captures the peak fall in FEV_1_ in EIB^+^ individuals (3)] participants completed a MDP(10). The MDP consists of 11 items evaluating sensory and affective dimensions of dyspnea, although affective dimensions were not assessed in the present study. The first item (A1) assesses the unpleasantness or discomfort of breathing on a scale ranging from 0 (“neutral”) to 10 (“unbearable”). The subsequent five items assess the intensity of sensory dimensions on a scale ranging from 0 (“none”) to 10 (“as intense as I can imagine”). The five items include, S1: my breathing requires muscle work or effort; S2: I am not getting enough air; S3: my chest and lungs feel tight or constricted; S4: my breathing requires mental effort or concentration; and S5: I am breathing a lot. Items were scored individually and as an “immediate perception domain score” (IPDS) calculated as the sum of A1 and S1–S5.

### Statistical analysis

Participants were grouped according to EVH test response (EIB^+^ or EIB^−^) and sex. Independent samples t-tests assessed between-group (EIB^+^ vs. EIB^−^; male vs. female) differences in baseline spirometry, average percentage of MVV achieved during EVH, peak fall in FEV_1_, and MDP items. Receiver operator characteristic (ROC) curves were determined for MDP items that differed between EIB^+^ and EIB^−^ groups. Stepwise multiple regression was performed using the peak fall in FEV_1_ as the dependent variable and MDP items as candidate predictors. Mixed model repeated measures ANOVA assessed the effects of time (baseline and 3, 10, 15, and 20-min post-EVH test) on FEV_1_, with a between-subjects factor of EIB diagnosis (EIB^+^ vs. EIB^−^). Significant main and interaction effects were followed by independent samples *t*-tests at each measurement point. Within-group changes in FEV_1_ were assessed using one-way repeated measures ANOVA followed by Tukey's post-hoc test. A Cochran Q test followed by a McNemar post-hoc test assessed differences in the number of EIB^+^ and EIB^−^ diagnoses based on each FI criteria. Pearson's correlation coefficient was used to determine the relationship between selected variables. Statistical significance was set at *P* < 0.05, except for the McNemar test, which was set at *P* < 0.0083. For significant differences, 95% confidence intervals are presented. Effect sizes are presented as Cohen's *d*. Data were analyzed using IBM SPSS Statistics V26.0 and presented as mean ± SD unless indicated otherwise.

## Results

**Figure 1:**
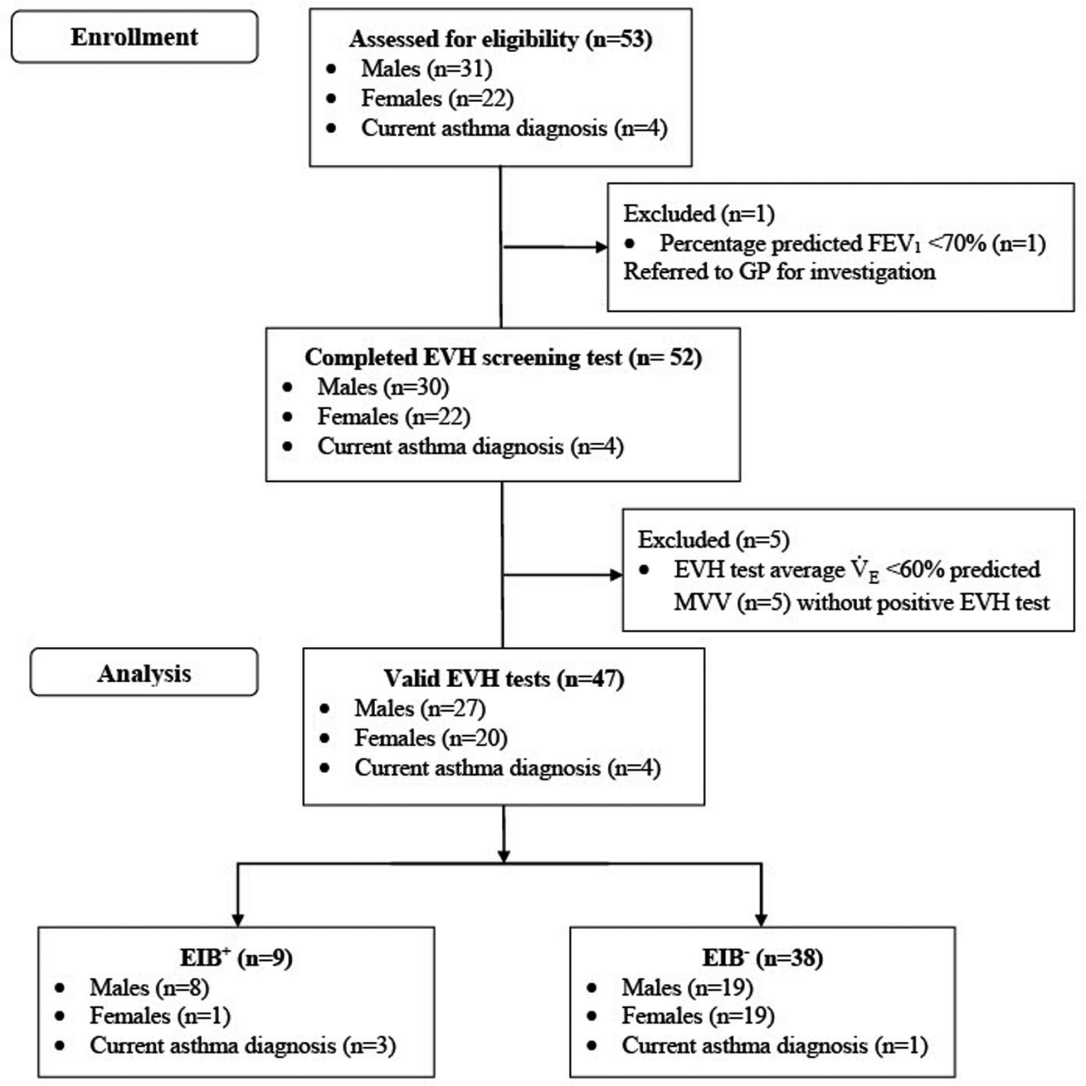
Participant flow diagram. FEV_1_, forced expiratory volume in 1 s; EVH, eucapnic voluntary hyperpnea; MVV, maximal voluntary ventilation.

One participant had a baseline FEV_1 _< 70% of predicted and therefore could not perform an EVH test. Five participants did not achieve an average ${\dot{V}}_{E}\geq 60\%$ MVV and not experience a ≥10% fall in FEV_1_ from pre-to-post-EVH test. One of these five participants accepted the invitation to repeat the EVH test on a separate day. During their second EVH test, this participant was again unable to achieve an average ${\dot{V}}_{E}\geq 60\%$ MVV and did not experience a ≥10% fall in FEV_1_ from pre-to-post-EVH test. Accordingly, all five participants were excluded from further analysis. Therefore, 47 participants (male = 27; female = 20) completed a valid EVH test (Figure 1). Of this cohort, 4 (9%) had a current GP diagnosis of asthma and none had a current EIB diagnosis.

### Baseline spirometry

Baseline spirometry (Table 1) was not different between EIB^+^ and EIB^−^ groups (*P* = 0.273–0.816; *d* = 0.09–0.41). FEV_1_ (% predicted) was lower in males (91 ± 10%) than females (97 ± 9%) [*P* = 0.032; 95% CI (−12, −0.6%); *d* = 0.63]. FVC (% predicted) was lower in males (94 ± 10%) than females (100 ± 10%) [*P* = 0.050; 95% CI (−11, −0.0004%); *d* = 0.58].

**Table 1:** Baseline pulmonary function for EIB^+^ and EIB^−^ groups, and male and female groups irrespective of EIB diagnosis. Values represent percentage of the predicted value.

	EIB^+^ (*n* = 9)	EIB^−^ (*n* = 38)	Male (*n* = 27)	Female (*n* = 20)
FEV_1_ (%)	91 ± 11	94 ± 10	91 ± 10^a^	97 ± 9
FVC (%)	95 ± 9	97 ± 11	94 ± 10^a^	100 ± 10
FEV_1_/FVC (%)	95 ± 9	98 ± 7	97 ± 8	98 ± 7
PEF (%)	97 ± 15	98 ± 12	96 ± 13	100 ± 12
FEF_25%–75%_ (%)	84 ± 25	93 ± 20	88 ± 22	96 ± 19

EIB, exercise-induced bronchoconstriction; EVH, eucapnic voluntary hyperpnea; FEV_1_, forced expiratory volume in 1 s; FVC, forced vital capacity; PEF, peak expiratory flow; FEF_25%–75%_, forced expiratory flow from 25 to 75% of FVC. Data are mean ± SD.

^a^ Difference between male and females (*P* ≤ 0.05).

### Spirometry after the EVH test

**Figure 2:**
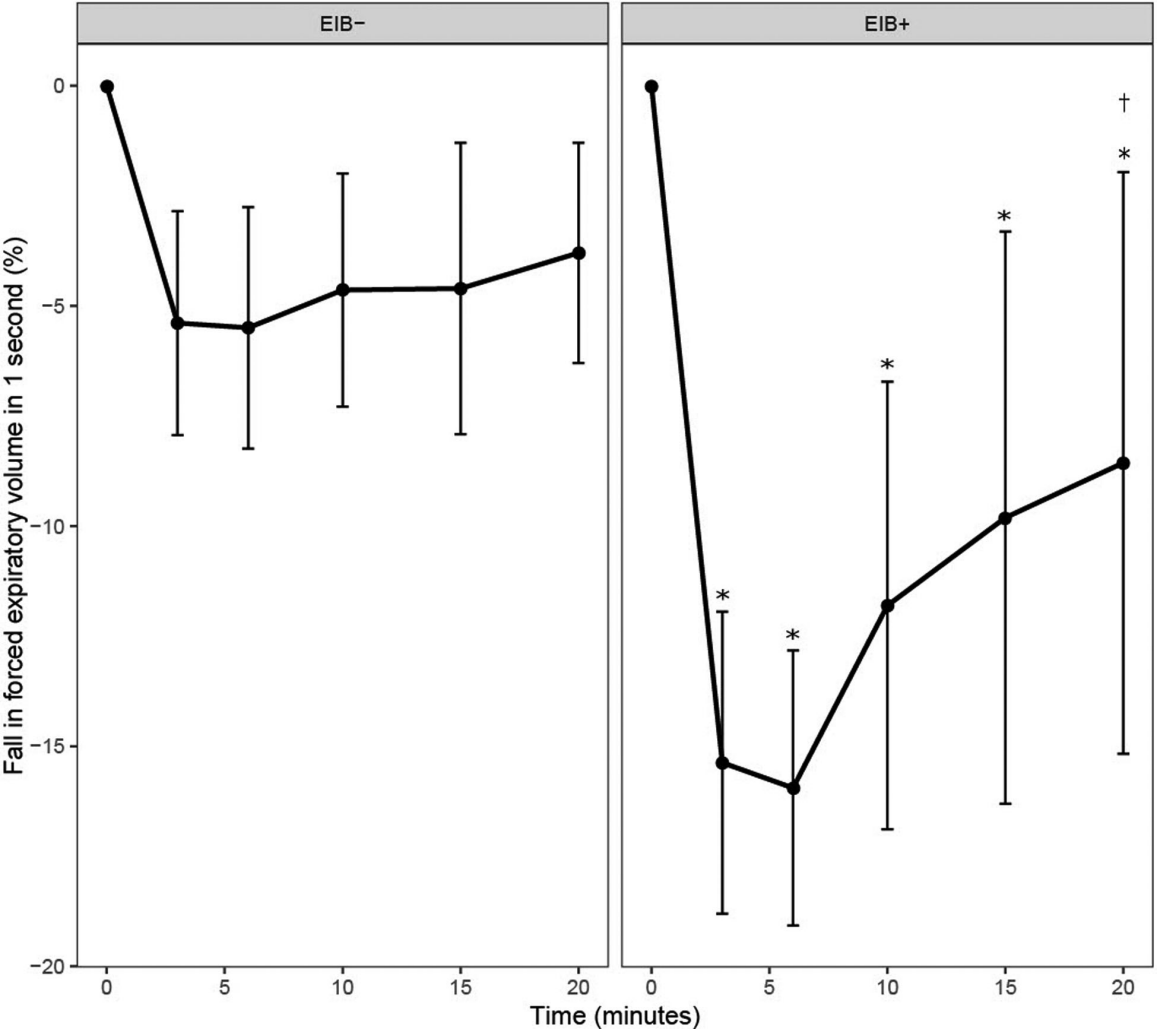
Fall in forced expiratory volume in 1 s (FEV_1_) from baseline (0) following the eucapnic voluntary hyperpnea test in EIB^+^ and EIB^−^ groups. *Different from baseline in EIB^+^ group (*P* ≤ 0.045). ^†^Different from 3- and 6-min post-EVH in the EIB^+^ group (*P* ≤ 0.002).

For the percentage fall in FEV_1_, there was a main effect of time and a group × time interaction (*P* ≤ 0.001). The fall in FEV_1_ was greater in the EIB^+^ group than the EIB^−^ group at all-time points after EVH (*P* ≤ 0.001; *d* = 1.2–2.1). In the EIB^+^ group, FEV_1_ was below baseline throughout recovery (*P* ≤ 0.006; *d* = 1.35–1.88). In the EIB^+^ group, peak falls in FEV_1_ were observed at 3-min (*n* = 4), 6-min (*n* = 2), 10-min (*n* = 2), and 15-min (*n* = 1) after EVH (Figure 2). By design, the peak fall in FEV_1_ was greater in the EIB^+^ (−18 ± 3%) than EIB^−^ (−7 ± 3%) group [*P* < 0.001; 95% CI (−14, −10%); *d* = 2.23]. The prevalence of EIB^+^ for all participants, males, and females, was 19%, 30%, and 5% respectively. Of the EIB^+^ participants 6/9 (66%) did not have a previous diagnosis of EIB or asthma. Three of the four athletes (75%) with a prior diagnosis of asthma were EIB^+^.

### Average minute ventilation during the EVH test

**Figure 3:**
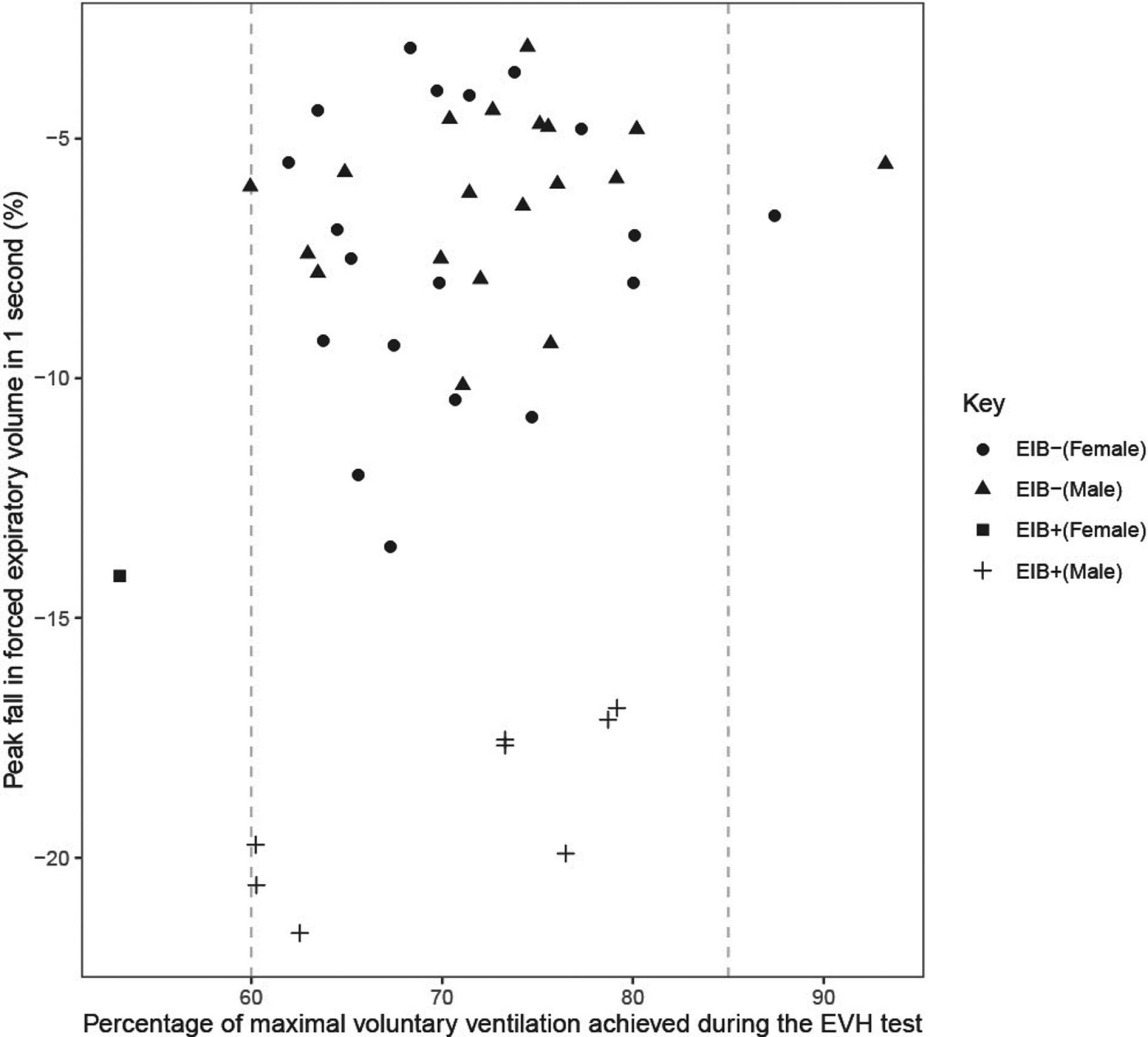
Peak fall in forced expiratory volume in 1 s (FEV_1_) from pre to post eucapnic voluntary hypernea (EVH) test in relation to the percentage of maximal voluntary ventilation (MVV) achieved during the EVH test. Vertical dashed line indicates the 60% and 85% of predicted MVV targets for participants during the EVH test.

Of the 52 participants completing an EVH test, six achieved less than 60% of predicted MVV, one of which was EIB^+^. Average ${\dot{V}}_{E}$ during the EVH test ranged from 53%–93% of predicted MVV in the 47 participants that completed a valid EVH test. Average ${\dot{V}}_{E}$ was not different between EIB^+^ (69 ± 10% MVV) and EIB^−^ (72 ± 7% MVV) groups (*P* = 0.246; *d* = 0.43), or between males (72 ± 8% MVV) and females (71 ± 7% MVV) (*P* = 0.492; *d* = 0.19). The average ${\dot{V}}_{E}$ during the EVH test was not related to the peak fall in FEV_1_ (*r* = 0.22, *P* = 0.147) (Figure 3).

### Effect of diagnostic criteria on EIB prevalence

The prevalence of EIB^+^ differed between FI criteria [*χ*^2^ (3) = 14.818, *P* = 0.002] (Table 2). Prevalence was greater under FI_10%−NORM_ than FI_ATS_ (*P* = 0.004), but no differences were found between FI_ATS_ and FI_≥10%_, or FI_≥10%_ and FI_10%−Norm_ (*P* ≥ 0.063).

**Table 2:** Prevalence (%) of EIB^+^ based on three different fall index (FI) criteria for all athletes (*n* = 47), males (*n* = 27), and females (*n* = 20).

	All	Male	Female
FI_ATS_	19 (9/47)	30 (8/27)	5 (1/20)
FI_≥10%_	30 (14/47)	33 (9/27)	25 (5/20)
FI_10%−Norm_	38 (18/47)^a^	44 (12/27)	30 (6/20)

^a^ Different from FI_ATS_ (*P* = 0.004). Ratios of EIB^+^/total group number within each group are shown in brackets.

### Dyspnea symptoms after the EVH test

**Figure 4:**
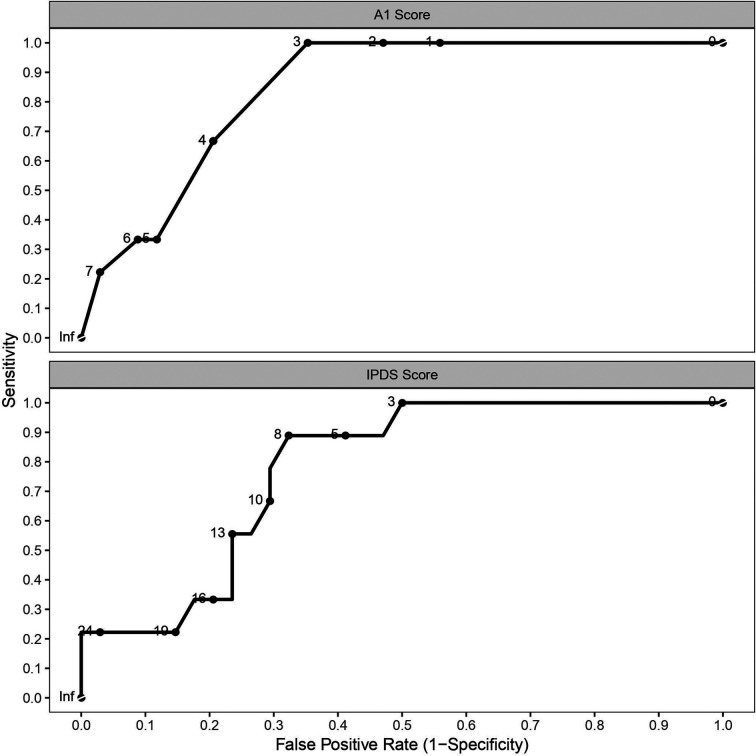
Receiver operating characteristics curves for multi-dyspnea profile items A1 (unpleasantness or discomfort of breathing) and IPDS (sum of all questions).

Of the 47 participants completing a valid EVH test, 43 successfully completed the MDP. Of these, 9 were in the EIB^+^ group. The IPDS (sum of all questions) was higher in the EIB^+^ (15 ± 9) than the EIB^−^ (6 ± 8) group [*P* = 0.031; 95% CI (2, 15); *d* = 1.1]. A1 (unpleasantness or discomfort of breathing) was higher in the EIB^+^ (5 ± 2) than the EIB^−^ (1 ± 2) group [*P* = 0.001; 95% CI (1, 4); *d* = 0.72]. Similarly, S3 (my chest and lungs feel tight or constricted) was higher in the EIB^+^ (3 ± 3) than EIB^−^ (1 ± 2) group [*P* = 0.014; 95% CI (0, 4); *d* = 1.4]. S1, S2, S4 and S5 items were not different between groups (*P* = 0.060–0.824; *d* = 0.09–0.92). ROC analyses produced significant area under the curves for A1 [AUC = 0.843; *P* ≤ 0.001; 95% CI (0.727, 0.959)], IPDS [AUC = 0.778; *P* = 0.011; 95% CI (0.635, 0.92)] scores but not S3 scores [AUC = 0.706; *P* = 0.055; 95% CI (0.496, 0.916)] (Figure 4). The maximal combined sensitivity and specificity cut-off for detecting EIB corresponded to scores of A1 = 3 (sensitivity = 1; specificity = 0.63; positive prediction value = 43%; negative prediction value = 100%), IPDS = 7 (sensitivity = 0.89; specificity = 0.66; positive prediction value = 42%; negative prediction value = 96%), and S3 = 3 (sensitivity = 0.67; specificity = 0.77; positive prediction value = 43%; negative prediction value = 90%). The peak fall in FEV_1_ after EVH correlated negatively with IPDS (*r* = −0.31; *P* = 0.044) and A1 (*r* = −0.45; *P* = 0.003) scores but not S3 scores (*r* = −0.28; *P* = 0.065). Multiple regression analysis of the peak fall in FEV_1_ revealed item A1 to be the only contributing factor.

## Discussion

### Main findings

The main findings of the present study were fourfold: (I) prevalence of EIB in British university field hockey athletes was 19% and greater in males (30%) than females (5%); (II) out of the nine participants identified as having EIB, six (66%) did not have a previous diagnosis of EIB or asthma and were untreated despite having a mean peak fall in FEV_1_ of 18%; (III) prevalence of EIB ranged from 19%–38% depending on the diagnostic FI criteria used; and (IIII) contemporaneous dyspnea symptoms (measured shortly after the EVH test) were higher in EIB^+^ participants and were sensitive in detecting an EIB diagnosis.

### Prevalence of EIB

The present study comprised, to date, the largest cohort of field hockey athletes screened for EIB. The prevalence of EIB in our cohort of field hockey athletes (19%) supports that EIB is prevalent in competitive athletes (3–5). Interestingly, none of the nine EIB^+^ athletes had a formal diagnosis of EIB. Three had a current GP diagnosis of asthma along with prescribed asthma maintenance and reliever therapy. Between 40%–90% of individuals with a physician diagnosis of asthma are estimated to have EIB (17). Similarly, we found 75% of participants with a previous diagnosis of asthma to be EIB^+^. Conversely, six of nine EIB^+^ athletes (66%) had no previous diagnosis of asthma and EIB and were untreated. These findings support previous screening studies in elite athletes which uncover a significant amount of EIB^+^ athletes with no prior diagnosis (3). These findings highlight the importance of screening athletes for EIB to ensure they receive appropriate treatment. Adequate treatment is important to facilitate athletic performance and reduce the risk of life-threatening bronchoconstriction.

EIB prevalence in the present study was lower than reported in other mixed sex university athletic populations (39%–43%) (4, 5). This is due, in part, to previous studies using FI_≥10%­­­_ to diagnose EIB, which is less conservative than the FI_ATS_ criteria and therefore results in higher prevalence rates (9). Indeed, using FI_≥10%_ in the current study yielded a 30% prevalence rate which is more comparable to previous reports (3–5, 18). Previous studies (4, 5) also screened for EIB in athletes from different sports, which affects prevalence rates. Compared to the present study, a higher prevalence of EIB (38%), based on FI_ATS_, has been reported in 21 elite hockey athletes (sex not reported) (3). Higher prevalence may be explained, in part, by higher training volumes performed by elite hockey athletes, which exacerbates stress on the airways and thereby increase the risk of developing EIB (19). Compared with the present cohort, the elite hockey athletes also achieved a higher % of predicted MVV during EVH (79% vs. 71% in the present study); however, a relationship between the % of predicted MVV achieved during EVH and the subsequent fall in FEV_1_ was not observed in the present study nor in the study of elite hockey athletes (3).

Of the 52 athletes completing an EVH test, six athletes (12%) were unable to achieve the ventilation requirements for a valid EVH test (≥60% predicted MVV) (12), including one EIB^+^ athlete. This was despite the investigators providing extensive encouragement and coaching for participants to achieve their target ${\dot{V}}_{E}$ during EVH tests. Previous reports have shown 4% of elite athletes and 21% of recreationally active non-athletes are unable to achieve ≥60% predicted MVV during the EVH test (3, 6), suggesting that valid EVH tests are more likely in elite athletes compared to non-elite athletes and non-athletes. In the present study, only two participants were able to equal or exceed their EVH test target ${\dot{V}}_{E}$ (≥85% predicted MVV) during the EVH test. This target ${\dot{V}}_{E}$ during EVH is often prescribed for testing in athletes (12), however this may not be attainable by most non-elite athletes. Due to issues with some participants achieving their ${\dot{V}}_{E}$ target during an EVH test, classifying individuals with borderline responses can be problematic. For example, in the present study, five athletes had ≥10% drop in FEV_1_ at only one time point post-EVH, which classifies them as EIB^−^ under the ATS guidelines. Borderline responses, particularly those with a ${\dot{V}}_{E}<60\%$ of predicted MVV, should thus be interpreted with care and additional EVH testing may be necessary to facilitate an accurate diagnosis (20). Moreover, further research is needed to establish: (1) whether borderline responses have clinical relevance or implications for exercise performance, and (2) the extent to which borderline responses reflect true EIB, which could be explored by examining the response to a bronchodilator after the EVH test.

### Sex differences in EIB prevalence

In the present study, the prevalence of EIB in field hockey athletes was lower in females (5%) than males (30%). Similar EIB prevalence rates (based on FI_≥10%_) between male (42%) and female (38%) university athletes have been reported previously (4), although the cohort of 22 sports did not include field hockey, which might partly explain this discrepancy. It might also be explained, in part, by differences in EIB diagnostic criteria. In support, if the diagnosis of EIB in the present study is based on FI_≥10%_, the prevalence in males (33%) and females (25%) is more comparable. In contrast to the present study, the prevalence of EIB was higher in female (26%) than male (18%) elite winter sport athletes (21). An explanation for this discrepancy is unclear but might be related to sport-specific differences such as environmental conditions and/or the type of challenge (exercise vs. EVH). It is also noteworthy that EIB screening in females is confounded by fluctuations in sex hormones during the menstrual cycle, with peak falls in FEV_1_ following exercise challenge tests being 4.5% greater during the mid-luteal phase of the menstrual cycle in comparison with the mid-follicular phase (22). Although it remains to be empirically tested/confirmed, fluctuations in female reproductive hormones would be expected to have similar effects on EVH test responses. Therefore, a limitation of the present study, and previous studies investigating EIB prevalence in females, is that menstrual cycle phase and contraceptive therapies were not documented / controlled. Nevertheless, our results suggest that the prevalence of EIB, when diagnosed using FI_ATS_, is lower in female compared to male field hockey athletes.

### Dyspnea and EIB

Dyspnea symptoms, namely the “unpleasantness or discomfort of breathing” (A1), “my chest and lungs feel tight or constricted” (S3), and the sum of A1 and scores relating to the intensity of sensory dimensions (IPDS), were higher in EIB^+^ than EIB^−^ field hockey athletes. Interestingly, A1 and IPDS scores had a sensitivity and specificy for detecting EIB comparable to a methacholine challenge (23). Our findings contrast previous studies reporting that only ∼50% of EIB^+^ individuals report dyspnea symptoms and that such symptoms are not associated with EIB (3–5, 7, 18). However, these studies administered questionnaires with participants at rest and asymptomatic, rather than soon after an exercise or EVH challenge when EIB and symptoms are present. This is a limitation for two reasons: (I) symptom recall from previous habitual exercise would lack validity if the stimulus was insufficient to induce bronchoconstriction (e.g., exercise ${\dot{V}}_{E}<85\%$ MVV); and (II) retrospective self-report measures that rely on memory are poorly tied to momentary biological processes and their fluctuation (8). Therefore, a strength of the present study is that the MDP was administered 3–10 mins after the EVH test and was therefore more likely to connect momentary conscious dyspnea symptoms with the underpinning pathophysiology of EIB (8). This may explain why the MDP items IPDS and A1 were both associated with the post-EVH fall in FEV_1_ and were good predictors of EIB classification in the present cohort. These results therefore suggest that contemporaneous dyspnea symptoms are associated with EIB diagnosis. Interestingly, an A1 score of 3 had a 100% negative prediction value to the post-EVH fall in FEV_1_ and, therefore, from a practical perspective this offers a useful tool for coaches / practitioners and athletes to rule out the likelihood that EIB is/was present.

## Conclusion

In conclusion, the prevalence of EIB in university field hockey athletes was 19% and greater in males than females. Many of the athletes identified as having EIB had no previous diagnosis of EIB or asthma despite having significant reductions in FEV_1_ and scoring highly for dyspnea symptoms. The prevalence of EIB depends on the FI criteria employed; thus, previous studies that did not use FI_ATS_ may have overestimated the prevalence of EIB. Finally, contemporaneous dyspnea symptoms were associated with EIB, had high negative prediction value, and may therefore offer a useful tool in ruling out the presence of EIB.

## Data Availability

The raw data supporting the conclusions of this article will be made available by the authors, without undue reservation.
